# Blood-Brain Barrier Abnormalities Caused by HIV-1 gp120: Mechanistic and Therapeutic Implications

**DOI:** 10.1100/2012/482575

**Published:** 2012-02-01

**Authors:** Jean-Pierre Louboutin, David S. Strayer

**Affiliations:** Department of Pathology, Anatomy and Cell Biology, Thomas Jefferson University, 1020 Locust Street Room 255 Philadelphia, PA 19107, USA

## Abstract

The blood-brain barrier (BBB) is compromised in many systemic and CNS diseases, including HIV-1 infection of the brain. We studied BBB disruption caused by HIV-1 envelope glycoprotein 120 (gp120) as a model. Exposure to gp120, whether acute [by direct intra-caudate-putamen (CP) injection] or chronic [using SV(gp120), an experimental model of ongoing production of gp120] disrupted the BBB, and led to leakage of vascular contents. Gp120 was directly toxic to brain endothelial cells. Abnormalities of the BBB reflect the activity of matrix metalloproteinases (MMPs). These target laminin and attack the tight junctions between endothelial cells and BBB basal laminae. MMP-2 and MMP-9 were upregulated following gp120-injection. Gp120 reduced laminin and tight junction proteins. Reactive oxygen species (ROS) activate MMPs. Injecting gp120 induced lipid peroxidation. Gene transfer of antioxidant enzymes protected against gp120-induced BBB abnormalities. NMDA upregulates the proform of MMP-9. Using the NMDA receptor (NMDAR-1) inhibitor, memantine, we observed partial protection from gp120-induced BBB injury. Thus, (1) HIV-envelope gp120 disrupts the BBB; (2) this occurs via lesions in brain microvessels, MMP activation and degradation of vascular basement membrane and vascular tight junctions; (3) NMDAR-1 activation plays a role in this BBB injury; and (4) antioxidant gene delivery as well as NMDAR-1 antagonists may protect the BBB.

## 1. Introduction

The blood-brain barrier (BBB) protects the brain by limiting the ability of molecules and cells from the blood to enter the CNS. The BBB is composed of brain capillary endothelial cells, interconnected with special intercellular tight junctions [1]. Along with astrocyte end feet, pericytes, basal lamina, and neurons, brain capillary endothelial cells comprise the neurovascular unit, which is important in maintaining the immune-privileged nature of the CNS and in regulating cellular transmigration [1].

Breach of BBB occurs in many diseases and, depending on the situation, may magnify the damage caused by the initial insult. Recently, it was suggested that BBB disruption mediates some of the tissue damage that accompanies human immunodeficiency virus-1 (HIV-1) infection of the brain, and so facilitates viral entry into the CNS [2]. In fact, as survival with chronic HIV-1 infection improves, the number of people harboring the virus in their CNS, where it is largely impervious to highly active antiretroviral therapeutic drugs (HAART), increases. Thus, the prevalence of HIV-associated neurocognitive disorder (HAND) continues to rise, and less fulminant forms of HAND such as minor neurocognitive/motor disorder (MCMD) have become more common than their more fulminant predecessors, and their presence remains a significant independent risk factor for AIDS mortality [3–6].

It is not clear how HIV-1 first enters the CNS. However, once the virus has entered the CNS, compromised BBB integrity may facilitate further viral entry into the CNS and magnify CNS injury [2]. BBB compromise is associated with neurocognitive impairment, and the combination of elevated plasma viral load and BBB compromise may increase the risk for development of HIV-associated dementia (HAD) [7]. Evidence of serum-protein leakage across the BBB has been demonstrated in the brains of HAD patients [8], and accumulation of serum proteins in subcortical neurons and glia is more common in HIV-1-positive patients with dementia than in those who lack cognitive impairment [9]. Brains of patients who died with HIV-1 encephalitis (HIVE) demonstrated absence or fragmentation of occludin and ZO-1, two important structural proteins of tight junctions, but there were no such changes in brains from patients HIV-seronegative controls or from HIV-1-infected patients without encephalitis [10].

In HIV-1 gp120-transgenic mice, expression of gp120, HIV-1 envelope glycoprotein, leads to extravasation of albumin and increased numbers of vessels immunostained for inter cellular adhesion molecule-1 (ICAM-1) and vascular cell adhesion molecule-1 (VCAM-1), as well as immunoreactivity for substance P at the endothelial cell surface [11, 12]. It has been reported that circulating gp120 alters BBB permeability in HIV-1 gp120 transgenic mice [13].

HIV-1 impairs CNS function in many ways [14]. Neurons themselves are rarely infected by HIV-1, and neuronal damage is probably mostly indirect. HIV-1 infection of resident microglia, periventricular macrophages and some astrocytes, leads to increased production of cytokines, such as IL-6, IL-1*β* and TNF-*α*, and chemokines such as MCP-1 [15]. Virus shedding or cytopathic effects in virus-infected cells result in release of gp120 protein and peptide products into the serum as well as the brain. Within the brain, infected microglia and macrophages can release HIV-1 proteins, several of which are neurotoxins, including nonstructural protein Tat and envelope (Env) protein gp120.

Besides examining human tissues, animal models have been used to study brain injury due to HIV-1 infection [16–19]. There are no perfect models for HAND. Several animal systems have been used to study the pathogenesis of HIV-1-induced neurological disease. Many of them are based on other lentiviruses (i.e., simian immunodeficiency virus infection of macaques, feline immunodeficiency virus infection of cats, Visna-Maedi virus infection in sheep) [20–22]. However, only small percentages of animals develop neurological manifestations in these models and the costs for using these species may be high. Transgenic expression of gp120 in mice has been studied [23], but the gp120 in that model is mainly expressed in astrocytes whereas in humans HIV-1 chiefly infects microglial cells. Other models based on introduction of HIV-infected macrophages into the brains of SCID mice have been proposed, but they suffer from the fact of human macrophages delivered into a murine brain [24].

We [18, 25] and others [16, 17] have used model systems in which recombinant gp120 or, Tat, proteins are directly injected into the striatum. The neurotoxicity of such recombinant proteins is highly reproducible and can be used as an interesting tool for testing novel therapeutic interventions. Administration of recombinant proteins is useful in understanding the effects of HIV-1 gene products, and so their individual contribution to the pathogenesis of HAND.

Gp120 is a potent neurotoxin, causing neurotoxicity in the picomolar range. Gp120 has been detected in the serum of HIV-infected patients [26]. The env mRNA encoding gp120 is also elevated in the brains of patients with HIV encephalitis [27]. It has been shown that gp120 is rapidly internalized in neurons, leading to apoptosis [28]. Attempts to detect gp120 either in brains of gp120 transgenic mice [23] or in HIV-1 autopsy brains have rarely been successful [29]. The rate of gp120 degradation in autopsy brains has not been reported yet. Gp120 is easier to detect when its expression in the brain is protracted [19]. In other terms, gp120 immunodetection is difficult, possibly due to technical factors. The absence of gp120 immunoreactivity does not preclude a role for gp120 in neuronal apoptosis, particularly at picomolar ranges and/or because of interactions with other HIV-1 neurotoxins (i.e., Tat). For example, concentrations of gp120 can be reduced by 10–20-fold in the presence of subtoxic concentrations of Tat to cause synergistic neurotoxicity [29]. Gp120 also causes synergistic neurotoxicity with glutamate [29, 30]. Moreover, gp120 at low doses can stimulate the production of cytokines from uninfected microglia and astrocytes.

To develop a model of acute exposure to gp120, different concentrations of gp120 were previously tested *in vitro* and *in vivo* [25, 31]. However, HIV-1 infection of the brain is a chronic process, and its study would benefit from a model system allowing longer-term exposure to HIV-1 gene product. This is in part the reason why we developed experimental models of chronic HIV-1 neurotoxicity based on recombinant SV40 (rSV40) vector-modified expression of gp120 [19] or Tat, in the brain.

In this review, we focused on BBB disruption caused by HIV-1 envelope glycoprotein 120 (gp120) as a model. Our studies have used both acute and chronic exposure to HIV-1 gp120, by respectively administering either recombinant protein or a SV40 viral vector that delivers ongoing gp120 expression. We report that gp120-induced disruption of the BBB occurs via lesions in brain microvessels, matrix metalloproteinases activation, and degradation of the vascular basement membrane and vascular tight junctions. Activation of the receptor for NMDA (NMDAR-1) plays a role in this BBB injury. Finally, antioxidant gene delivery using rSV40 vectors and NMDAR-1 antagonists may protect the BBB.

Thus, the effects of HIV-1 gp120 exposure, either acute or protracted, on BBB and their consequences are examined in the present review.

## 2. HIV-1 gp120 Directly Damages CNS Blood Vessels

We tested the effect of HIV-1 gp120 on BBB integrity by assessing BBB leakiness after exposure to gp120 [32]. After injection of different concentrations of gp120 (100, 250, and 500 ng/microl) into the caudate putamen (CP), terminal deoxynucleotidyl transferase-mediated nick end labeling (TUNEL) assays were performed from 6 h to 14 days, to detect neuron apoptosis. There were extremely rare TUNEL-positive cells when the CP was injected with saline (negative control). At all doses of gp120, TUNEL-positive cells peaked one day after the injection. The number of TUNEL-positive cells was significantly higher for 500 ng gp120. The dose of 500 ng gp120 was, unless otherwise specified, used in further studies [31].

**Figure 1:**
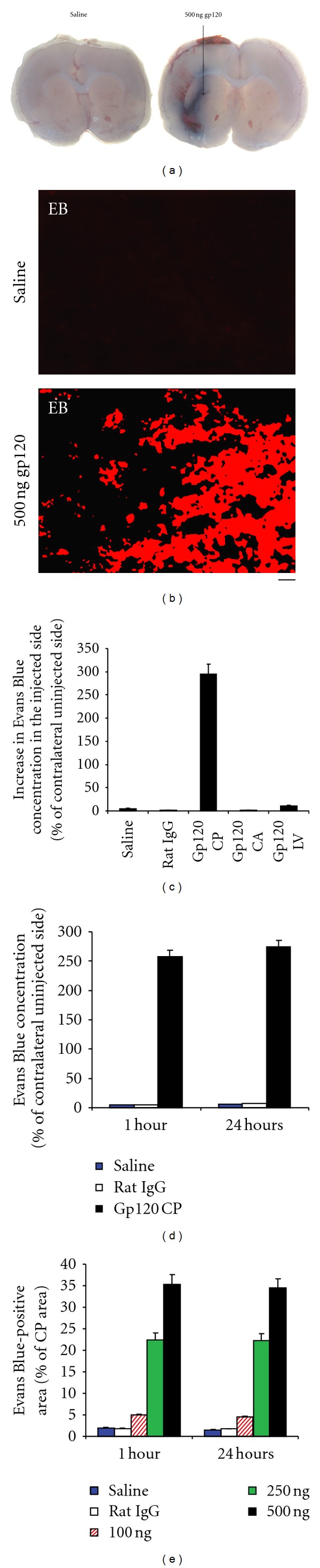
Injection of gp120 into the rat caudate-putamen (CP) induced extravasation of Evans Blue (EB). (a) EB was injected intravenously before injection of gp120. Gp120 (500 ng in 1 *μ*l saline) was injected stereotaxically into the rat CP using coordinates obtained from the rat brain atlas of Paxinos and Watson (1986). Controls received saline or rat IgG. One day later, extravasation of EB was seen in the CP. (b) Fluorescence corresponding to EB extravasation was observed by microscopy in the injected CP. (c) Spectrophotometric measurements representing the specific absorbance of EB at 620 nm showed that EB levels were higher in the CP injected with gp120 compared to CPs injected with saline or rat IgG. Furthermore, injection of gp120 into the carotid artery (CA, 2000 ng gp120) or the lateral ventricle (LV, 2000 ng gp120) did not increase EB levels in the CP. (d) EB levels in the CP were almost similar 1 hour and 1 day after gp120 injection. (e) Relationship between the concentrations of gp120 and the levels of EB. Bar: (b) 100 *μ*m.

Thus, rats were injected with the vital dye Evans Blue (EB) shortly before stereotaxic injection of 500 ng gp120 in 1 *μ*l saline into the CP. Presence of EB outside the CNS vascular system was used as an indicator of BBB damage and consequent leakage of vascular contents into the brain. EB extravasation was detected as blue color in brain tissue and as a red fluorescent signal in the injected CP. EB leakage was seen as early as 15 min after gp120 injection. EB extravasation increased 1 hour after gp120 injection, and remained intense until 24 hours. No such leakage was observed on the contralateral side or when saline or a control protein, rat IgG, was injected instead of gp120 (Figures 1(a) and 1(b)). No significant leakage of EB was observed when gp120 was injected into the carotid artery (CA) or in the lateral ventricle (LV). After injection of gp120 into the CP, much higher EB concentrations were measured in the injected CP, compared to the uninjected side as well as to saline- or rat IgG-injected CPs. There was no statistical difference between both sides in the rats injected with gp120 into the CA or in the LV (Figure 1(c)). Higher EB concentrations were measured as early as 1 hour after injection of gp120 (Figure 1(d)). Finally, there was a relationship between the amount of gp120 injected into the CP and the EB concentration measured in the same structure (Figure 1(e)). 

## 3. Gp120 Is Directly Toxic to Brain Endothelial Cells

Gp120 is cytotoxic to human umbilical vein endothelial cells [33], as well as those from the brain and lung [34]. Human brain microvascular endothelial cells (HBMECs) do not express CD4, but do have cell surface CCR5 and CXCR4 chemokine receptors, which are coreceptors for HIV-1 [14]. Exposure of HBMECs to gp120 derived from macrophage (CCR5)- or lymphocyte (CXCR4)-tropic viruses decreased BBB tightness, increased permeability and enhanced monocyte migration across *in vitro* BBB models [35]. Anti-CCR5 antibodies inhibited [Ca^2+^]_i_ release induced by gp120 derived from CCR5-tropic HIV-1, and the CXCR4 antagonist AMD3100 partially blocked [Ca^2+^]_i_ release caused by gp120 derived from CXCR4-tropic HIV-1. Moreover, CCR5 antibodies and inhibitors of myosin light chain kinase or protein kinase C (PKC) blocked gp120-induced permeability and enhanced monocyte migration *in vitro* [35]. Thus, gp120 can cause dysfunction of the BBB via receptor mediated [Ca^2+^]_i_ release and PKC pathways leading to cytoskeletal alterations and increased monocyte migration [35].

Gp120 induced apoptosis in cultured human endothelial cells [36], and gp120 and Tat have been reported to induce oxidative stress in brain endothelial cells [37]. Gp120 can be directly toxic to such cells in culture, and reduces their production of the tight junction protein, occludin [34, 35]. However, despite these *in vitro* studies, the effects of gp120 injection on the cerebral vascular endothelium in intact animals have not yet been described [16–18].

We therefore investigated the effects of gp120 on brain endothelial cells *in vitro* and *in vivo *[32]. We first tested whether gp120 could induce apoptosis of these cells in culture. The optimal concentrations of HIV-1 gp120 to elicit apoptosis *in vitro* was determined using NT2-neurons for neurons and human brain microvascular endothelial cells (HBMECs) for endothelial cells. Cells were incubated with 0, 0.1, 1, 10, 100, and 200 ng/mL of recombinant soluble gp120. Apoptotic bodies were analyzed using TUNEL. The intensity and frequency of TUNEL-positive cells increased with increasing gp120 doses, up to 200 ng/mL. Higher concentrations of gp120 caused cells to detach and so were not further studied. Thus, 200 ng/mL of gp120 was used in *in vitro* studies.

**Figure 2:**
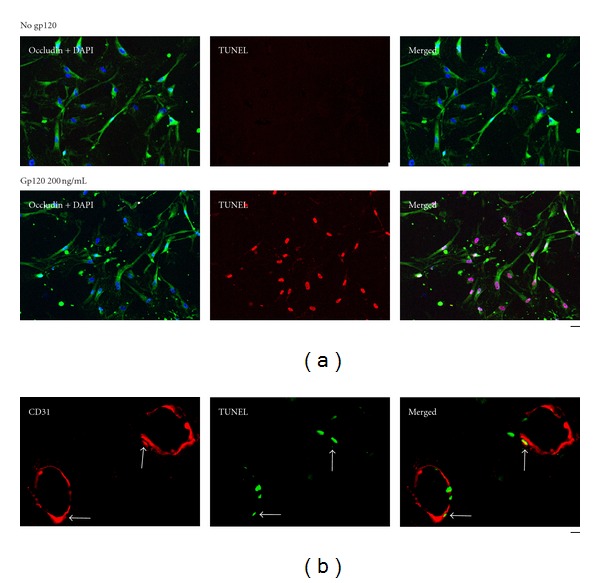
Gp120 is toxic to endothelial cells. (a) Human brain endothelial cells immunostained for occludin and incubated with 200 ng/mL gp120 became apoptotic. The nucleus was immunostained by 4′,6-diamidino-2-phenylindole (DAPI). Apoptotic cells were assayed by TUNEL. Numerous TUNEL-positive cells were observed after treatment with gp120 while almost no apoptotic cells were seen in cultures that were not incubated with gp120 (*P* < 0.001 serum-free media (control) versus 200 ng/mL gp120 in serum-free media for 24 hours). Almost all occludin-positive cells were TUNEL-positive for 200 ng/mL gp120. Control with buffer only and no enzyme showed no TUNEL staining (not shown). (b) Cryostat sections of rat CP injected with 500 ng gp120 1 hour earlier immunostained for CD31 and processed for TUNEL assay. Some of the endothelial cells, immunostained for CD31, are apoptotic and TUNEL-positive (arrows). Bars: (a) 50 *μ*m; (b) 60 *μ*m.

Cultured human brain microvascular endothelial cells (HBMECs) were treated with serum-free medium (control) or 200 ng/mL gp120 in serum-free medium for 24 hours. They were then assayed by TUNEL for apoptosis, and immunostained for occludin (a tight junction protein in brain endothelial cells). Numerous TUNEL-positive cells were observed after treatment with 200 ng/mL gp120. Almost all occludin-positive cells were TUNEL-positive for 200 ng/mL gp120. Few apoptotic cells were seen in controls (Figure 2(a)).

To apply these studies to living animals, we injected rats with 500 ng HIV-1 BaL gp120 in 1 *μ*l saline (or, control, saline or rat IgG only), stereotaxically into the CP. One hour after injection of gp120 into the CP, some endothelial cells (identified as immunopositive for CD31) were TUNEL-positive (Figure 2(b)). No apoptotic cells were observed after injection of control preparations [25]. Thus, gp120 induces apoptosis of endothelial cells.

## 4. Gp120 Increases MMP Production

The basal lamina of the BBB contains extracellular matrix (ECM) molecules, such as laminin, type IV collagen, and fibronectin. Most of these are substrates for a family of neutral proteases called matrix metalloproteinases (MMPs), especially MMP-2 and MMP-9. MMP-2 and MMP-9 are classified as gelatinases [38]. MMPs contribute to interactions between cells and their matrix, allowing movement and shape changes important in CNS development and neuronal plasticity. They are also implicated in opening the BBB, invasion of neural tissue by blood-derived immune cells and direct cellular damage such as occurs in diseases of the central and peripheral nervous systems [39–48]. Increased MMP production may injure the BBB, at least in part through their proteolytic activity at the tight junctions of brain endothelial cells and the BBB basal lamina. Evidence suggests that MMPs contribute to HIV-associated brain injury by 3 main mechanisms: breakdown of the BBB, induction of neuronal death, degradation of myelin [49].

### 4.1. Gelatinolytic Activity Is Activated by gp120 Administration

**Figure 3:**
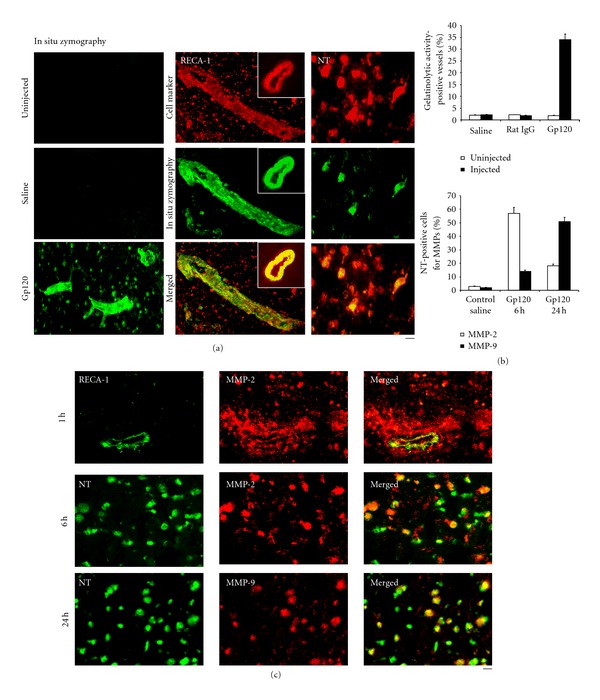
Injection of gp120 into the rat CP induced upregulation of matrix metalloproteinases (MMPs). (a) Frozen cryostat sections of CP injected with 500 ng gp120 and stained by *in situ* zymography. Left column: the fluorescent product was observed in blood vessel-like structures and in cells. No gelatinolytic activity was seen in the controlateral uninjected side or in CPs injected with saline. There was a colocalization between *in situ* zymography and RECA-1 and Neurotrace (NT), a marker of neurons (resp., middle and right columns). (b) Top: numbers of vessels exhibiting gelatinolytic activity were increased in CPs injected with gp120. Bottom: increase in the numbers of MMP-2- and MMP-9-positive neurons in CPs injected with gp120. (c) Colocalization between MMP-2, MMP-9, NT, and RECA-1 in CPs injected with gp120. Bar: (a) left column: 100 *μ*m, middle column: 70 *μ*m, left column: 30 *μ*m; (c) first row: 50 *μ*m, 2nd row: 40 *μ*m, 3rd row: 40 *μ*m.

We tested proteolytic activity by evaluating cleavage of the fluorogenic substrate DQ gelatin in frozen tissue sections [50]. We found that local gelatinolytic activity was increased within 30 min after gp120 administration. Gelatinolytic activity was still evident by this technique for at least 60 min after injection of gp120 into the CP in vascular structures and cells. Almost no gelatinolytic activity was seen in the contralateral uninjected side or after injecting either saline or saline containing rat IgG instead of gp120. Nearly all such activity was blocked by preincubating tissue sections with a zinc chelator (1,10-phenanthrolin) for 20 min (Figure 3(a)). The number of vessels showing gelatinolytic activity was also increased in CPs injected with gp120, compared to the uninjected contralateral side and compared to recipients of control preparations (Figure 3(b)). 

To identify cell types responsible for gp120-induced gelatinolytic activity, we combined *in situ* zymography with immunostaining for different cell markers. Most gelatinolytic activity was within neurons positive for NeuroTrace (NT) [51, 52], and, extracellularly, in blood vessel walls, as identified by their expression of rat endothelial cell antigen (RECA-1). Gelatinolytic activity also colocalized, but much less frequently, within endothelial cells (immunopositive for CD31). There was very rare activity within astrocytes (immunopositive for GFAP; Figures 3(a) and 3(b)).

### 4.2. MMP-2 and MMP-9 Are Expressed Early in the Injured Striatum after gp120 Injection

Since MMP-2 and MMP-9 are the major tissue proteases capable of targeting microvascular proteins, we assessed the time courses of their expression following intra-CP injection of gp120. MMP-2 and MMP-9 were increased after gp120 injection into the CP: increased MMP-2 was first detected 30 min to 1 hour after inoculation and had largely subsided by 24 hours. The time course of MMP-9 was different. It was first detected 6 hours after injection, peaked at d1, then decreased at d2. Neither enzyme was detected in the contralateral (uninjected) side or if saline was substituted for gp120. The spatial relationship between EB extravasation and MMP-2 was established by colocalization studies. Increased MMP-2 was detected in the area of vascular leakage, as evidenced by EB positivity, within 1 hour of gp120 administration. Gp120 induced MMP-9 localized mainly in neurons, and, rarely, in astrocytes. MMP-2 was observed within blood vessel walls and was mainly seen in neurons, rarely in astrocytes and exceptionally in microglial cells. It was not detected in control CPs injected with saline (Figure 3(c)).

Thus, increased enzymatic activity corresponding to activation of MMP-2 and MMP-9 was observed within 30 min of exposure to gp120. This activity and the MMP-2/MMP-9 proteins were both mainly detected in neurons, in vessel matrices, and in endothelial cells. Localization of both MMPs and gelatinolytic activity within neurons has also been reported by others [40, 44, 53].

HIV-1 viral proteins have been implicated in activation of MMPs. Tat increased release of MMP-1 and MMP-2 in neuron cultures [54] and, in combination with basic FGF, activated MMP-2 and membrane-type-1 matrix metalloproteinase in endothelial cells [55]. Stable expression of gp120 *in vitro* and *in vivo* increased MMP-2 activity in the brain [56]. After injection of gp120 into the lateral ventricle, levels of MMP-2 and MMP-9 increased rapidly; prior administration of an MMP inhibitor reduced consequent neuronal apoptosis [57]. In rapidly progressing simian immunodeficiency virus-infected monkeys, levels of MMP-9 correlated with motor and cognitive deficits [58].

## 5. Degradation of the Vascular Basement Membrane and Vascular Tight Junctions after gp120 Injection: Relationship with MMPs Activation

**Figure 4:**
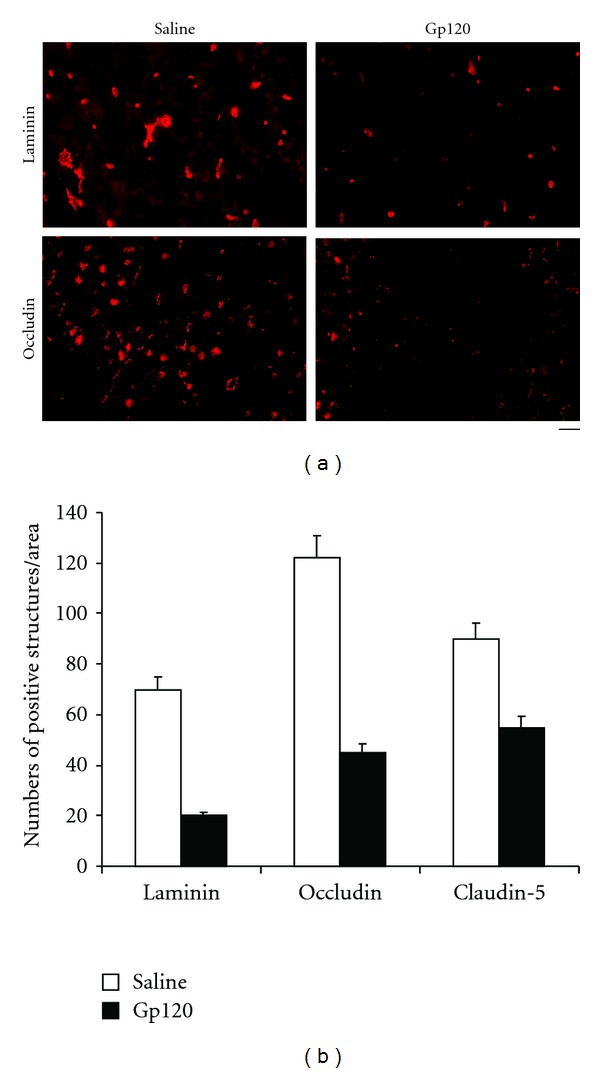
Gp120 induced a degradation of vascular basement membrane and vascular tight junctions. (a) After intra-CP injection of 500 ng gp120, fewer laminin- (a basement membrane protein) and occludin-(a tight junction protein) positive structures were seen. (b) Graph showing a significant reduction of laminin-, occludin-, and claudin-5-(another tight junction protein) positive structures in CPs injected with 500 ng gp120. Bar: (a) 60 *μ*m.

MMPs cleave basement membrane proteins, for example, laminin. Quantitative methods were used to assess the numbers of laminin-positive structures. Within 30 min of gp120 injection, MMP-2 colocalized with laminin, and by 6 hours there was a significant reduction in the number of laminin-positive structures in the injected CP. Numbers of laminin-positive structures were unaltered in the contralateral (uninjected) side or when saline was substituted for gp120 (Figure 4(a)). Similarly, the vascular tight junction proteins, claudin-5 and occludin, were significantly decreased in CPs injected with gp120 compared to controls. Claudin-5-positive structures were quantitated and were most reduced in areas of vascular leakage, suggesting that gp120-elicited enzymatic destruction of basement membrane and tight junctions proteins causes impaired BBB integrity (Figure 4(b)).

## 6. Gp120 Reduces the Number of Brain Microvessels

**Figure 5:**
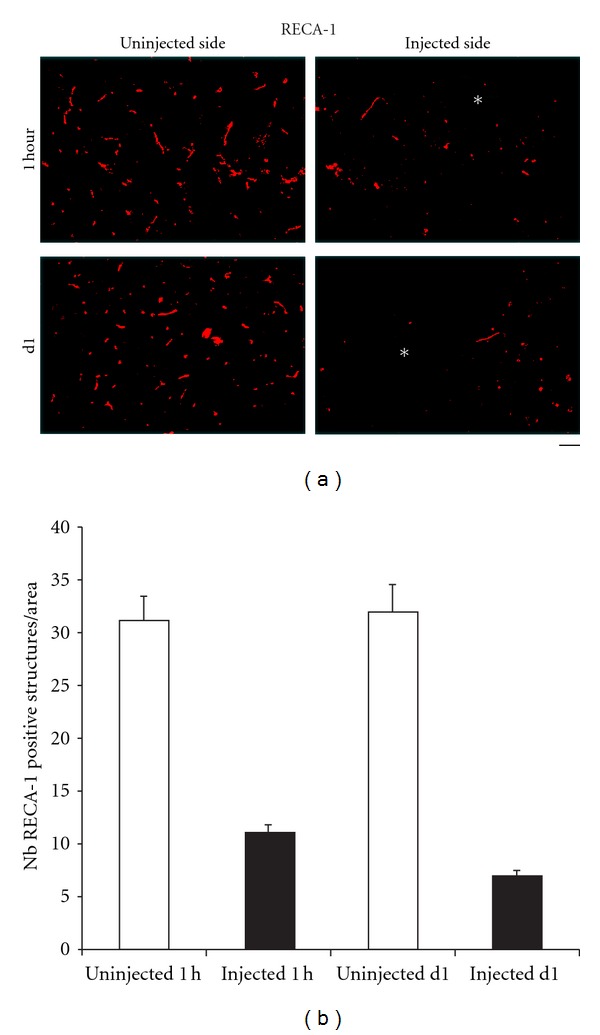
Reduction in the number of brain microvessels after gp120 injection. A decrease in the number of RECA-1-(a marker of microvessels) positive structures was seen after injection of 500 ng gp120 into the rat CP. This effect was observed as early at 1 hour after the injection. Bar: 60 *μ*m.

Quantitative methods were used to assess brain vessel density by using VCAM-1 and RECA-1 as markers of microvessels. VCAM-1- and RECA-1-positive structures were counted manually on the injected and uninjected sides in the entire CP of animals injected with either gp120 or saline, in at least 5 consecutive sections using a computerized imaging system (Image-Pro Plus, MediaCybernetics, Bethesda, MD). In all cases, the final number was an average of results measured in the different sections. We found that injection of gp120 led to a significant decrease in the number of brain microvessels, compared to controls (uninjected contralateral side), within one hour (Figure 5).

## 7. NMDAR-1 Activation Plays a Role in gp120-Induced BBB Injury

**Figure 6:**
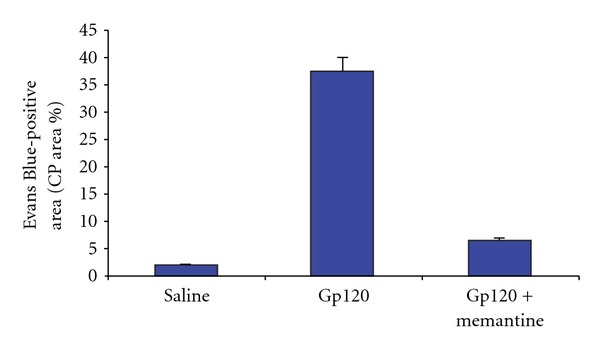
NMDAR-1 activation plays a role in gp120-induced BBB injury. The potential involvement of NMDA in the BBB disturbances induced by gp120 was studied using memantine, a NMDA receptor antagonist. Injection of memantine (30 mg/kg) before (i.v.) injecting EB, followed by intra-CP gp120, limited the extent of the EB-positive area.

N-methyl-D-aspartate (NMDA) has been reported to upregulate the proenzyme form of MMP-9 and to increase MMP-9 proteolytic activity [59]. The potential involvement of NMDA in the BBB disturbances induced by gp120 was studied using memantine, an NMDA receptor antagonist. Memantine has been shown to reduce pathological activation of MMP-9 in some conditions. Injection of memantine (30 mg/kg) before (i.v.) injecting EB, followed by intra-CP gp120 limited the extent of the EB-positive area (Figure 6). Memantine pretreatment also mostly protected vascular structures from gp120 toxicity. The partial but not complete recovery of BBB leakage by memantine might be related to the dose of memantine we used. However, other mechanisms may play a role too.

## 8. Gp120 *In Vivo* Induces Oxidative Stress in Endothelial Cells: Gene Delivery of SOD1 and GPx1 Protects the BBB from Acute gp120-Related Injury

Activation/upregulation of MMPs may involve reactive nitrogen and oxygen radicals (RNS and ROS, resp.) such as NO and superoxide [43, 45]. ROS are important in the pathogenesis of HIV-induced CNS injury [60], and increased by HIV-1 gp120 and Tat in brain endothelial cells [61].

**Figure 7:**
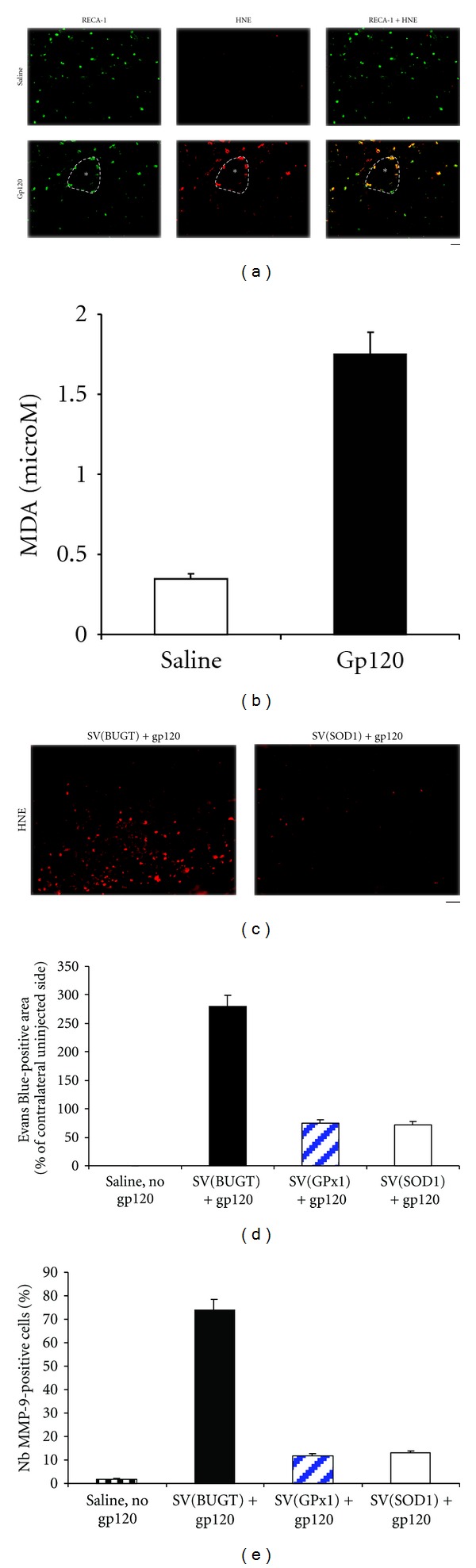
Role of oxidative stress in gp120-induced BBB disruption. (a) Cryostat sections of rat CP injected 1 hour earlier with saline or 500 ng gp120 were immunostained for HNE, a marker of lipid peroxidation, and RECA-1, a marker of endothelial cells. HNE immunoreactivity was seen in the CPs injected with gp120 and colocalized with RECA-1 while no HNE immunostaining was detected in CPs injected with saline. (b) Malondialdehyde (MDA) levels were increased in the CPs injected with 500 ng gp120. (c) Prior gene delivery of antioxidant enzymes into the CP before injection of gp120 decreased the number of HNE-positive cells when compared with injection of the vector control SV(BUGT). (d) Gene delivery of antioxidant enzymes by SV40-derived vectors in the CP 1 month before the injection of gp120 into the same structure mitigated the extent of BBB breakdown after intra-CP injection of gp120, as demonstrated by spectrophotometry measurements of EB. (e) Fewer MMP-9-positive cells were observed with prior gene delivery of antioxidant enzymes into the CP before injection of gp120. SV(BUGT) was used as a control vector. Bar: (a) 80 *μ*m; (c) 80 *μ*m.

Lipid peroxidation by ROS can be assayed as formation of hydroxynonenal (HNE) [60]. To identify cell populations undergoing gp120-induced lipid oxidation, we performed double immunostaining for cell lineage markers and HNE, a marker of lipid peroxidation after administering gp120 into the CP. Within 1 hour of gp120 injection, brain vasculature contained HNE-positive cells, which expressed the endothelial cell marker RECA-1. Gp120 thus induces early lipid peroxidation in endothelial cells (Figure 7(a)). HIV-1 envelope glycoprotein also causes oxidative damage in neurons and astrocytes (not shown). Lipid peroxidation in brains injected with gp120 was measured by quantitating malondialdehyde (MDA) levels. MDA was significantly higher in CPs injected with gp120 than in control CPs (Figure 7(b)).

We used SV40-derived vectors, carrying antioxidant enzymes, Cu/Zn superoxide dismutase (SOD1), or glutathione peroxidase (GPx1), respectively, SV(SOD1) and SV(GPx1). rSV40s were employed in the current study because they transduce cells in G0 with high efficiency [62–65]. SV(BUGT), carrying human bilirubin-uridine 5′-diphosphate-glucuronosyl-transferase, was used as a control vector [66]. We previously reported that SOD1 and GPx1 are expressed in the CP after injection of SV(SOD1) and SV(GPx1) in rats [25, 31, 67], and in Rhesus macaques [68].

Intracerebral injection of rSV40s carrying cDNAs for these antioxidant enzymes significantly protected neurons from apoptosis and other consequences of subsequent injection of HIV-1 gp120 at the same location [18, 25, 31, 69]. Vector administration into the lateral ventricle (LV) or the cisterna magna, particularly if preceded by mannitol i.p., protects from intra-CP gp120-induced neurotoxicity comparably to intra-CP vector administration [31, 70].

Fewer HNE-positive cells were observed after prior gene transfer of antioxidant enzymes in the CP before injecting gp120, suggesting that lipid peroxidation was decreased by gene delivery of antioxidant enzymes (Figure 7(c)).

Prior gene delivery of antioxidant enzymes, SV(SOD1) and SV(GPx1), into the CP before injection of gp120 into the same structure limited BBB breakdown caused by gp120. Morphometric analysis documented that antioxidant gene delivery decreased gp120-induced BBB damage, as measured by EB-leakage: the size of the EB-positive area and spectrophometric measurement of EB concentration (Figure 7(d)). Finally, intra-CP gene delivery of either of the antioxidant enzymes before injection of gp120 decreased the number of MMP-9-positive cells when compared with injection of the vector control SV(BUGT), Figure 7(e).

 Thus, we have shown, both here and previously, that antioxidant enzyme gene delivery protected from neuronal apoptosis and lipid peroxidation caused by HIV-1 envelope gp120 [18, 25, 31, 70]. Also, reduction in gp120-induced oxidative stress by antioxidant gene delivery decreased MMP-9-positive cells and protected the BBB from disruption [50]. However, antioxidant gene delivery induced a partial but not complete recovery of BBB leakage. It might be related to an insufficient transgene expression. On the other hand, other molecules taking place in HIV-induced BBB leakage might play a role too.

## 9. BBB Disturbances after Injection of SV(gp120) into the CP

**Figure 8:**
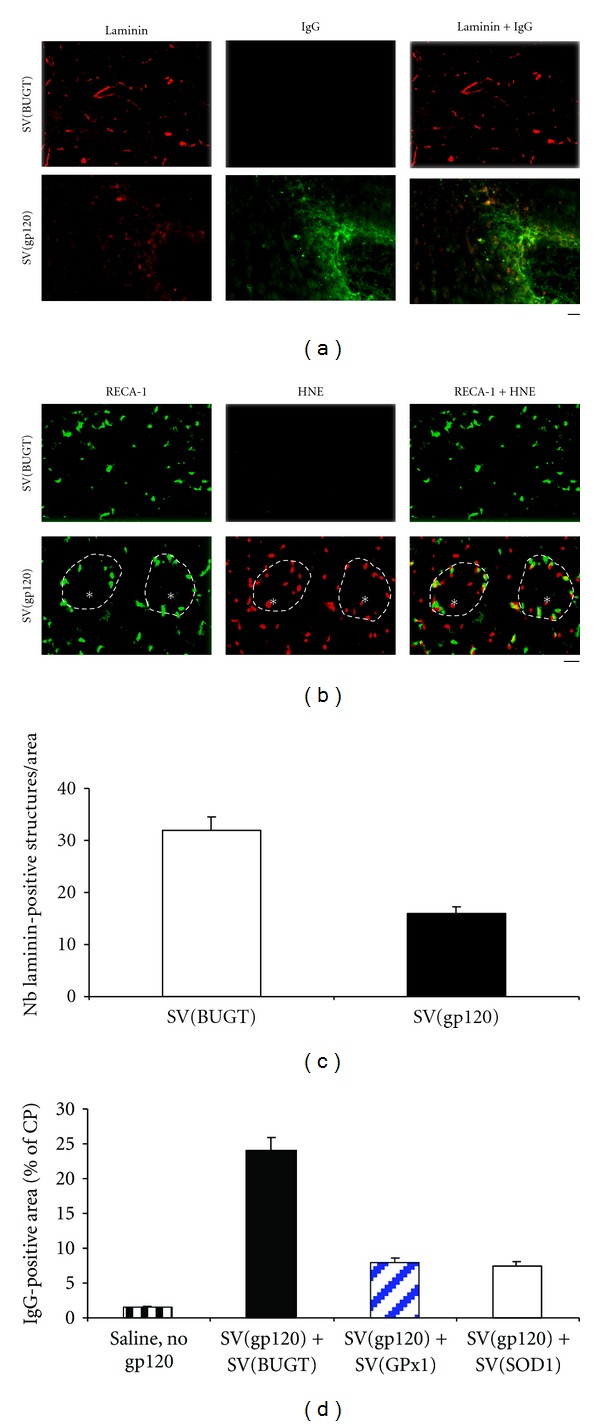
Injection of SV(gp120) into the CP increased BBB permeability. (a) Cryostat sections of the CP from rats injected with SV(gp120) or a control vector, SV(BUGT), at the level of the striatum, were immunostained for IgG (to evaluate leakage of plasma protein through the BBB) and laminin. Seven days after injection of SV(gp120) into the CP, significantly less laminin-positive structures were seen, particularly in the areas of IgG accumulation, while laminin immunostaining was normal and no IgG leakage was observed after injection of SV(BUGT). (b) Sections of rat CPs injected with SV(gp120) or a control vector, SV(BUGT), were immunostained for HNE, a marker of lipid peroxidation, and RECA-1, a marker of endothelial cells. Immunostaining for HNE was seen in the CPs injected with SV(gp120) and colocalized with RECA-1, while no HNE immunoreactivity was detected in CPs injected with SV(BUGT). (c) Injection of SV(gp120) induced a decrease in the number of laminin-positive structures. (d) Prior gene delivery of antioxidant enzymes into the CP before injection of SV(gp120) decreased the extent of IgG-positive area after intra-CP injection of SV(gp120). Bar: (a) 60 *μ*m; (b) 50 *μ*m.

As exposure to HIV-1 in HIV/AIDS patients is protracted, we asked whether BBB injury could be seen in a model system of more chronic CNS exposure to gp120. In this model system for HIV-1 Env-induced neurotoxicity, SV(gp120) injection into the CP leads to chronic expression of gp120 in microglia and neurons, ongoing apoptosis of these cell types, neuronal loss, and oxidative stress [19]. In many respects, the consequences of rSV40-delivered gp120 expression in this system resemble the pathologic and biochemical alterations observed in neuroAIDS. Rats were injected with SV(gp120) intra-CP, which leads to continued gp120 production by transduced cells. These animals were then studied over time for evidence of BBB injury. Using immunohistochemical detection of serum IgG leakage from the blood vessels into the brain substance as a marker of vascular permeability, we measured the areas of IgG accumulation 7 days after intra-CP administration of SV(gp120). The area positive for IgG was significantly larger when SV(gp120) was injected than when the control vector, SV(BUGT), was used, or compared to the contralateral side. Fewer laminin-positive structures were seen in the SV(gp120) injected side, Figure 8(a).

We then examined brains of SV(gp120) for lipid peroxidation in endothelial cells, using HNE as a marker of lipid peroxidation, and RECA-1 to identify endothelial cells. Immunostaining for HNE was seen, and colocalized with RECA-1, in CPs injected with SV(gp120), but not in CPs injected with SV(BUGT) (Figure 8(b)).

To study the relationship between IgG leakage and vascular breakdown, we studied brain microvessels immunostaining for laminin, a basement membrane protein. SV(gp120) injection greatly decreased laminin-positive structures, compared to SV(BUGT) and to the uninjected contralateral side, which effect was particularly marked in areas of IgG accumulation (Figure 8(c)). Results at later time points (2 and 4 weeks) were similar to those observed at 1 week. These data indicate that ongoing expression of gp120, delivered by SV(gp120), increased BBB permeability, and that this increase in vascular leakiness persisted as long as gp120 was being produced.

We then examined the effect of SV(gp120) treatment on levels of MMP-2 and MMP-9. At all time points tested, 1, 2, and 4 weeks, after injection of SV(gp120), MMP-2, and MMP-9 were increased. MMP-2- and MMP-9-positive cells were mainly neurons.

As was observed for acute exposure to recombinant gp120, prior gene transfer of antioxidant enzymes mitigated BBB damage caused by ongoing gp120 production following SV(gp120) injection. This was measured as extravasated IgG. Thus, the increased BBB permeability induced by continuing gp120 production can be reduced by overexpression of antioxidant enzymes (Figure 8(d)).

MMP-2 activity and protein were increased in C6 cells (stable transfectants for gp120) and in gp120-transgenic mouse brains [56]. In gp120 transgenic mice, Finco et al. quantitated the number of vessels with perivascular albumin and the number of ICAM-1- and VCAM-1-positive structures [11]. The number of vessels with perivascular albumin was significantly higher in transgenic mice, suggesting an increase in BBB permeability. However, the increased number of ICAM-1- and VCAM-1-positive structures contrast with our results obtained with laminin as a vessel marker. However, these markers do not recognize the same molecules on one side and on the other side the number of ICAM-1-positive structures can be increased during different processes, for example, during inflammation. An increase of ICAM-1 expression by human endothelial cells cells has been demonstrated *in vitro* after exposure to gp120 [71–73]. However, the degree of upregulation of ICAM-1 differed among the various human brain microvascular endothelial cells isolates [73].

In our system, gene delivery provides ongoing gp120 expression, as a model of production of HIV-1 Env by HIV-1-infected cells in neuroAIDS. It generates chronic vascular injury that resembles the BBB damage caused by HIV-1 infection.

## 10. Consequences of BBB Abnormalities for the Migration of HIV-1 Infected Blood Cells into the Brain

Diapedesis of monocytes from the blood into the brain is facilitated when MMPs target occludin, a tight junction molecule of CNS endothelial cells. Dendritic cell transmigration through brain microvessel endothelium is also affected by MMPs, as well as by MIP-1*α*, and is associated with occludin reorganization [1].

Compromise of BBB integrity has important consequences for the migration of HIV-1-infected blood cells into the brain. HIV-1 in the circulation can cross the BBB either as free virus or within infected immune cells [74, 75], and circulating free gp120 can cross the BBB [76]. In a tissue culture model of the human BBB, HIV infection increases leukocyte transmigration in response to the chemokine, CCL-2 (MCP-1). This is accompanied by disruption of the BBB, with enhanced permeability, reduction of tight junction proteins and increased MMP-2 and MMP-9 [77]. Thus, a vicious cycle is possible: HIV-1 infected monocytes enter the CNS through the microvasculature, produce HIV-1 proteins (gp120 and Tat) that damage the BBB directly and indirectly, and thereby allow increased immigration of HIV-1-infected cells, perpetuating the cycle.

We show here that gp120 injection reduces the number of VCAM-1-positive structures. VCAM-1 has been used in the present study as marker of microvessels. Previous studies showed an increase in the number of VCAM-1 and ICAM-1-positive brain vessels in the brain of HIV-1 gp120 transgenic mice [12]. These results were obtained using transgenic mice in which gp120 is expressed chronically in astrocytes while in humans HIV-1 infects mainly microglial cells. With injection of recombinant gp120, exposure is more acute and might reflect the direct action of gp120 on microvessels. Immunostaining for RECA-1, a marker of rat endothelial cells and microvessels, showed a reduction in RECA-1-positive structures that paralleled the ones of VCAM-1-positive microvessels. Similarly, a significant decrease in vascular density assessed by immunostaining for laminin was observed at early times and persisted through one month after gp120 injection, suggesting that even the acute effects of gp120 may be transient, there are also longer-term consequences of gp120 exposure on the vascular density.

 Antioxidant gene delivery and NMDAR-1 antagonists induced a partial but not complete recovery of BBB leakage. It suggests that other molecules might take place in HIV-induced BBB leakage. Among these molecules, a role for substance P has been suggested [11]. For example, modulation of substance P and its receptor (neurokinin 1 receptor, NK1-R) have been shown to block some unidentified molecular pathways leading to HIV-1-mediated BBB damage [78]. Substance P (SP), a pleotropic neuropeptide implicated in inflammation, depression, and immune modulation via interaction with its cognate receptor, the neurokinin 1 receptor (NK1-R), is produced by monocyte/macrophages. While the presence of NK1-R on neurons is well known, its role on cells of the immune system such as monocyte/macrophages is just beginning to emerge. The expression of SP and NK1-R and their relationship to SIV/HIV encephalitis (SIVE/HIVE) lesions and SIV-infected cells has been examined recently [79]. These studies demonstrated intense expression of SP and NK1-R in SIVE lesions, with macrophages being the principal cell expressing NK1-R. Additionally, studies of the functional role of SP as a proinflammatory mediator of monocyte activation and chemotaxis demonstrated that treatment of monocytes with SP elicited changes in cell-surface expression for CCR5 and NK1-R in a dose-dependent manner. Moreover, pretreatment with SP enhanced both SP- and CCL5-mediated chemotaxis. All of these findings suggest that SP and NK1-R are important in SIV infection of macrophages and the development of SIVE lesions.

 Other mechanisms have also been suggested. Stating that activation of MMP-9 is involved in HIV-1-induced disruption of the BBB, Huang et al. [80] hypothesized that peroxisome proliferator-activated receptor (PPAR)*α* or PPAR*γ* can protect against HIV-1-induced MMP-9 overexpression in brain endothelial cells (hCMEC cell line) by attenuating cellular oxidative stress and downregulation of caveolae-associated redox signaling. HIV-1stimulated activity of MMP-9 promoter was inhibited by mutation of AP-1 site 2 and both (but not individual) NF-*κ*B binding sites (g1389c and g1664c). PPAR overexpression, ERK1/2 or Akt inhibition, and silencing of cav-1 all effectively protected against HIV-1-induced MMP-9 promoter activity, indicating a close relationship among HIV-1-induced cerebrovascular toxicity, redox-regulated mechanisms, and functional caveolae. Such a link was further confirmed in MMP-9-deficient mice exposed to PPAR*α* or PPAR*γ* agonist and injected with the HIV-1 protein Tat into cerebral vasculature. Overall, these results indicate that ERK1/2, Akt, and cav-1 are involved in the regulatory mechanisms of PPAR-mediated protection against HIV-1-induced MMP-9 expression in brain endothelial cells. Moreover, down-regulation of MMP and proteasome activities seems to constitute a novel mechanism of PPAR-induced protections against HIV-induced disruption of tight junction proteins and brain endothelial cells [81].

## 11. Conclusions

We showed that both acute and chronic exposure to HIV-envelope gp120 disrupts the BBB by causing lesions in brain microvessels, which probably reflect gp120-induced MMP activation with consequent degradation of the vascular basement membrane and vascular tight junctions. All of these factors lead to loss of BBB integrity and enhanced leakage of vascular contents into the brain. NMDAR-1 activation plays a role in this BBB injury, as do reactive oxygen species. Antioxidant gene delivery and NMDA-R antagonists protect from gp120-induced BBB injury and so provide an intriguing potential avenue for developmental therapeutics. However, antioxidant gene delivery and NMDAR-1 antagonists induced a partial but not complete recovery of BBB leakage. It suggests that other molecules might take place in HIV-induced BBB leakage. Among these molecules, a role for substance P has been suggested [11]. For example, substance P antagonists and neurokinin, as well as PPAR*α* or PPAR*γ* have been shown to modulate HIV-induced disruption of the BBB.
